# Tumour response to TRK inhibition in a patient with pancreatic adenocarcinoma harbouring an *NTRK* gene fusion

**DOI:** 10.1093/annonc/mdz385

**Published:** 2019-11-18

**Authors:** E M O’Reilly, J F Hechtman

**Affiliations:** Memorial Sloan Kettering Cancer Center, New York, USA

**Keywords:** *NTRK* gene fusion, TRK fusion cancer, tropomyosin receptor kinase inhibition, pancreatic adenocarcinoma, larotrectinib, selitrectinib

## Abstract

**Background:**

Although rare, *NTRK* gene fusions are known to be oncogenic drivers in pancreatic ductal adenocarcinoma (PDAC). We report the response of a metastatic *CTRC-NTRK1* gene fusion-positive PDAC to targeted treatment with the oral tropomyosin receptor kinase (TRK) inhibitor larotrectinib and the eventual development of resistance to treatment.

**Patient, methods and results:**

A 61-year-old woman presented with a 2.5-cm mass in the body of the pancreas and a 1.2-cm liver lesion on routine follow-up for endometrial cancer that was in complete remission. Liver biopsy confirmed a primary PDAC unrelated to the endometrial cancer. The patient was treated with gemcitabine, nab-paclitaxel and ADI-PEG 20 for 12 months until disease progression and toxicity emerged [best overall response (BOR): partial response (PR)]. The patient switched to a modified regimen of folinic acid, fluorouracil, irinotecan and oxaliplatin for 4 months until neuropathy occurred. Oxaliplatin was withheld until disease progression 6 months later (BOR: stable disease). Despite recommencing oxaliplatin, the disease continued to progress. At this time, somatic profiling of the liver lesion revealed a *CTRC-NTRK1* gene fusion. Treatment with larotrectinib 100 mg twice daily was commenced with BOR of PR at 2 months. The patient progressed after 6 months and was re-biopsied. Treatment was switched to the investigational next-generation TRK inhibitor selitrectinib (BAY 2731954, LOXO-195) 100 mg twice daily. After 2 months, the disease progressed and dabrafenibtrametinib combination therapy was initiated due to existence of a *BRAF-V600E* mutation. However, the cancer continued to progress and the patient died 2 months later.

**Conclusions:**

Targeted TRK inhibition with larotrectinib in PDAC harbouring a *CTRC-NTRK1* gene fusion is well tolerated and can improve quality of life for the patient. However, acquired resistance to therapy can emerge in some patients. Next-generation TRK inhibitors such as selitrectinib are currently in development to overcome this resistance (NCT02576431; NCT03215511).

## Key Message

*NTRK* gene fusions are known to be oncogenic drivers in pancreatic ductal adenocarcinoma (PDAC), thus providing a potential target for therapy with tropomyosin receptor kinase (TRK) inhibitors. This case report describes a patient with *CTRC-NTRK1* gene fusion-positive PDAC treated with larotrectinib, a selective TRK inhibitor, who subsequently developed resistance due to an acquired *BRAF* mutation.

## Background

The most common type of malignant cancer of the pancreas is pancreatic ductal adenocarcinoma (PDAC). More than one-half of PDAC cases are identified with locally advanced or metastatic disease at presentation, with metastases mainly found in the liver, lungs and peritoneum [1]. The overall survival across all stages of disease is 8%; this decreases to 3% in patients who present with advanced disease [1]. PDAC is characterised by several germline or acquired genetic mutations. In total, 90%–95% of PDAC tumours are found to harbour an oncogenic *KRAS* mutation; other frequent mutations are *TP53* (75%), *SMAD4* (22%), *CDKNA/B* (18%) [2] and *BRCA1/2* (4.6–8%) [3]. Although rare, *NTRK* gene fusions are also known to be oncogenic drivers in <1% of PDAC cases [4], thus providing a potential target for therapy. *NTRK* gene fusions occur in up to 1% of all solid tumours in both adult and paediatric patients and have been shown to be oncogenic drivers that are actionable with tropomyosin receptor kinase (TRK) inhibitors, such as larotrectinib [5–7].

## Patient, methods and results

**Figure 1.**
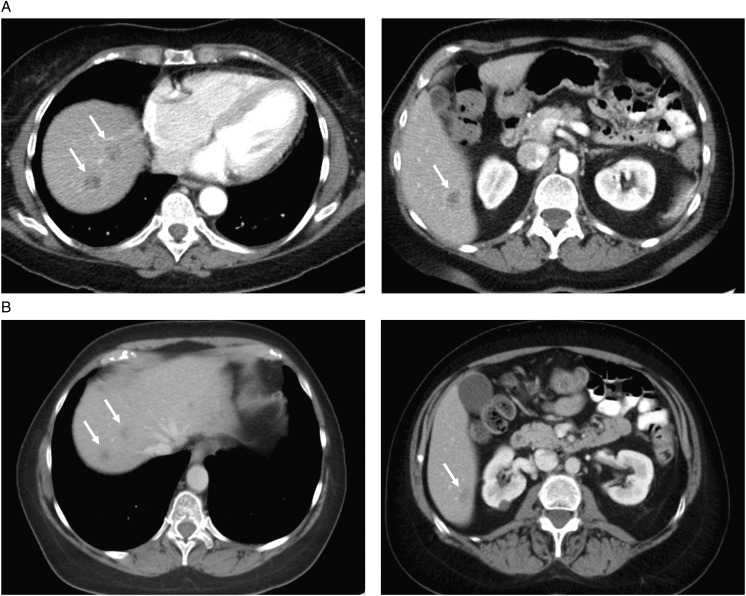
Computed tomography imaging. Computed tomography taken at (A) baseline before initiation of larotrectinib and (B) showing the best overall response of partial response to treatment with larotrectinib.

**Figure 2.**
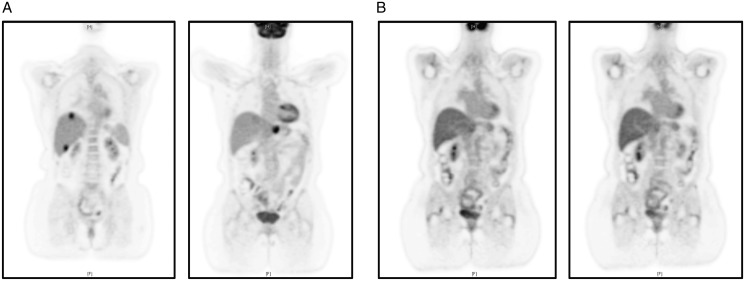
[^18^F]2-fluoro-2-deoxy-D-glucose positron emission tomography (FDG-PET) imaging. (A) FDG-PET imaging taken at baseline before initiation of larotrectinib. 2-fluoro-2-deoxy-D-glucose (FDG)-avidity is visible in the primary pancreatic tumour and liver metastases. (B) FDG-PET imaging showing the best overall response of partial response to treatment with larotrectinib. FDG-avidity in previously hypermetabolic pancreatic and liver lesions is resolved.

A 61-year-old woman with stage 1A, grade 1 endometrial cancer underwent a total abdominal hysterectomy with sentinel lymph node dissection followed by two rounds of intravaginal radiation and achieved complete remission without subsequent recurrence [8]. Seven months later, routine follow-up computed tomography imaging revealed a 2.5-cm mass in the body of the pancreas and a 1.2-cm liver lesion; positron emission tomography confirmed 2-fluoro-2-deoxy-D-glucose (FDG)-avidity in these areas (Figures 1A and 2A). Liver biopsy demonstrated a poorly differentiated adenocarcinoma that was CK7+/CK20–, consistent with a primary PDAC; this was morphologically different from the prior endometrial cancer. Germline profiling was negative for mutations in *BRCA1/2*, *PALB2*, *ATM* and DNA mismatch repair genes. Of specific note, there was insufficient tissue present for genomic profiling of the PDAC at this time; both the primary and metastatic tumour sites were very small and the yield was anticipated to be low.

The patient began treatment with gemcitabine, nab-paclitaxel and ADI-PEG 20 as part of a phase IB/II clinical trial, continuing this regimen for 12 months until disease progression/emerging toxicity (haemolytic uremic syndrome) occurred. The best overall response (BOR) during this period was partial response (PR). The patient was switched to mFOLFIRINOX (a modified regimen of FOLinic acid, Fluorouracil, IRINotecan, and OXaliplatin) and underwent eight cycles over 4 months; the BOR was stable disease. The patient experienced neuropathy and therefore oxaliplatin was withheld for the next 11 cycles until disease progression 6 months later; the BOR during this period was stable disease. Oxaliplatin was recommenced in the context of disease progression and the patient received two cycles of mFOLFIRINOX over 1 month; however, the disease continued to progress.

**Figure 3.**
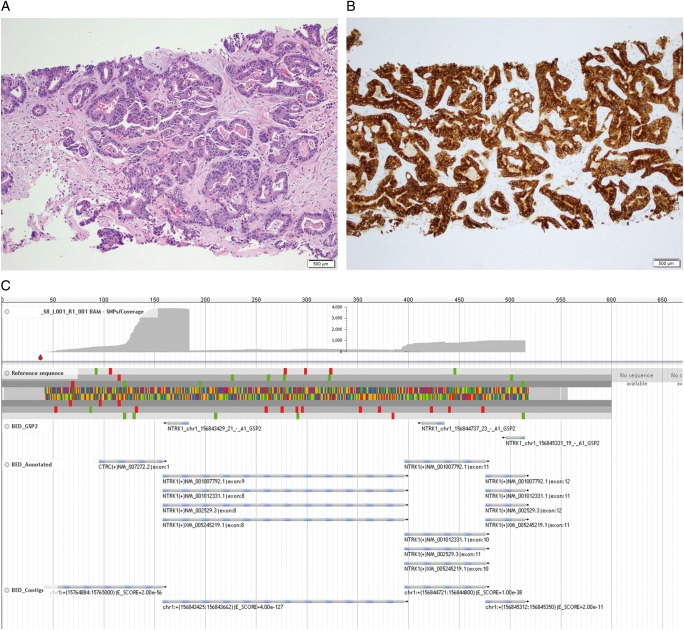
Liver biopsy analysis. (A) Haemotoxylin and eosin (H&E): A core biopsy of the patient’s liver mass demonstrated a moderately differentiated adenocarcinoma, morphologically compatible with pancreatobiliary origin (H&E, 100× original magnification). (B) TrkA immunohistochemistry (IHC): Immunohistochemical staining for TrkA (*NTRK1*) demonstrated diffuse, strong cytoplasmic expression (TrkA IHC, clone EP1058Y, Abcam, Cambridge, UK, 100× original magnification). (C) Archer^®^ software: Fusion analysis was carried out on the tumoral RNA with the MSK-IMPACT™ panel and demonstrated an in-frame fusion between *CTRC* (NM_007272) exon1 and *NTRK1* (NM_002529) exon8, including the kinase domain of *NTRK1* (JBrowse software).

A second liver biopsy was undertaken as there was now sufficient tissue to perform next-generation sequencing for somatic profiling. Liver biopsy analysis confirmed moderately differentiated liver adenocarcinoma which was morphologically and immunophenotypically compatible with pancreatic cancer (Figure 3A). Immunohistochemistry showed tumour cells that were CK7+ and negative for CK20, CDX2, PAX8, WT1, ER and PgR, supporting this diagnosis. TrkA immunohistochemistry further showed tumour cells strongly and diffusely positive for TrkA expression (Figure 3B). Somatic DNA profiling using Memorial Sloan Kettering-Integrated Mutation Profiling of Actionable Cancer Targets (MSK-IMPACT™; Memorial Sloan Kettering Cancer Center, New York, NY, USA) [9, 10] revealed an in-frame fusion between genes *CTRC* exon1 and *NTRK1* exon8. Additional molecular findings from MSK-IMPACT™ included gain of *SDHC*, deletion of *CDKN2B*, *CDKN2A* and *SMAD4*, an *ARID2* intragenic deletion, microsatellite stable status and a low tumor burden (0.9 mutation per megabase). Archer^®^ FusionPlex^®^ Custom Solid Panel with Anchored Multiplex PCR (AMP™; ArcherDX, Inc., Boulder, CO, USA)) [11] confirmed the presence and transcription of the in-frame *CTRC-NTRK1* gene fusion (Figure 3C and Table 1).

**Table 1.** NGS and Archer^®^ results pre- and post-larotrectinib treatment

	NGS pre-larotrectinib	Archer^®^ pre-larotrectinib	NGS at POD on larotrectinib
	Liver mass, right	Liver mass, right	Liver lesion
Clinically validated panel Somatic			
alterations	Negative	Negative	*BRAF* exon15 alteration
			(p. V600E [c.1799T>A])
Investigational panel			
Somatic alterations	*CTRC-NTRK1* gene rearrangement^a^	*CTRC-NTRK1* gene rearrangement^a^	*CTRC-NTRK1* gene rearrangement^a^
	(c.88: *CTRC*_c.850 + 45: *NTRK1*del)		(c.88: *CTRC*_c.850 + 46: *NTRK1*del)
	*SDHC* gain (1q23.3)^b^		
	*CDKN2B* deletion (9p21.3)		
	*CDKN2Ap16INK4A* deletion (9p21.3)		
	*CDKN2Ap14ARF* deletion (9p21.3)		
	*SMAD4* exon9 deletion		*SMAD4* exon9 deletion
	(p. R361_C363del [c.1081_1089delCGCTTTTGT])		(p. R361_C363del [c.1081_1089delCGCTTTTGT])
	*ARID2* rearrangement^c^		
	(c.419-2860_c.638-69del)		
			*ARAF* exon2 alteration
			(p. V21G [c.62T>G])
			*TBX3* exon7 alteration
			(p. G509A [c.1526G>C])

a The *CTRC-NTRK1* rearrangement is a deletion which results in the in-frame fusion of *CTRC* to *NTRK1* and includes the kinase domain of *NTRK1*. One of the breakpoints is within exon2 of *CTRC*.

b The *SDHC* copy number gain falls slightly below the cut-off criteria for amplification. Confirmatory testing by an alternate method is suggested, if clinically indicated.

c The *ARID2* rearrangement is an intragenic deletion of exon5. The functional significance is undetermined.

NGS, next-generation sequencing; POD, progression of disease.

Based on the identification of the actionable *CTRC-NTRK1* gene fusion, the patient was enrolled in NAVIGATE (NCT02576431), a phase II basket trial of the first-in-class oral selective TRK inhibitor larotrectinib [7], approved for the treatment of cancers harbouring *NTRK* gene fusions. The patient received larotrectinib at a dose of 100 mg b.i.d.; treatment was well tolerated. The BOR was PR at 2 months, with disease progression at 6 months. Imaging demonstrated resolution of FDG-avidity in previously hypermetabolic pancreatic and liver lesions (Figure 2A and 2B). Repeat liver biopsy was carried out at the time of disease progression. MSK-IMPACT™ revealed a new *BRAF-V600E* mutation, suggesting acquired resistance to larotrectinib mediated by mutation of the kinase domain of *BRAF* (Table 1). The patient was subsequently enrolled in a study with the next-generation TRK inhibitor selitrectinib (BAY 2731954, LOXO-195; 100 mg b.i.d.; NCT03215511) for patients with *NTRK* gene fusions who have progressed on a prior TRK inhibitor. After 2 months, best response of disease progression necessitated a switch in treatment and the patient initiated dabrafenib–trametinib combination therapy; however, the cancer continued to progress, and the patient died 2 months later.

## Discussion

This case is of special interest for several reasons. The patient presented with metastatic PDAC after a history of unrelated endometrial cancer; while there was no germline mutation to explain the tumour co-occurrence, ultimately, somatic profiling revealed a *CTRC-NTRK1* gene fusion that was therapeutically actionable in the PDAC. The patient received targeted therapy with larotrectinib that was well tolerated and led to a BOR of PR with excellent quality of life. The patient developed resistance to larotrectinib after 6 months which was associated with the emergence of an acquired *BRAF* mutation as a new oncogenic driver.

Despite initial durable responses to TRK inhibition therapy, it is expected that acquired resistance to therapy will ultimately emerge in a fraction of patients, often mediated by solvent-front mutations that directly interfere with binding by larotrectinib [12] or by the emergence of bypass mutations. On-target kinase domain mutations are the most common mechanism of acquired drug resistance to TRK inhibitors found in patients [13]. However, a mechanistic study of off-target resistance to TRK inhibitors recently reported by Cocco et al. [14] indicated that a subset of patients with gastrointestinal malignancies treated with TRK inhibitors may develop resistance due to MAP kinase pathway-activating genomic alterations. Exploration of molecular mechanisms of resistance used preclinical patient-derived xenograph (PDX) models established from the pre-treatment tumour of a patient with a *CTRC-NTRK1* gene fusion-positive PDAC who had an acquired resistance to larotrectinib. In these PDX models, prolonged treatment with larotrectinib resulted in the outgrowth of tumours with a newly acquired *BRAF-V600E* mutation, thus replicating the development of this bypass mutation in the clinical setting and in this case report. The frequency of resistance mutations in response to TRK inhibitor treatment in patients with TRK fusion cancer and the relative frequency of solvent-front versus bypass resistance mutations remains to be determined [14].

## Conclusion

In all, the patient lived for 14 months after an actionable gene fusion was identified, relatively late in the patient’s disease course. This present case illustrates the clinical benefit of larotrectinib in the treatment of patients harbouring *NTRK* gene fusions, as well as the mechanism behind acquired resistance to TRK inhibitors and the clinical implications of this resistance. 
